# PEEK with Reinforced Materials and Modifications for Dental Implant Applications

**DOI:** 10.3390/dj5040035

**Published:** 2017-12-15

**Authors:** Fitria Rahmitasari, Yuichi Ishida, Kosuke Kurahashi, Takashi Matsuda, Megumi Watanabe, Tetsuo Ichikawa

**Affiliations:** 1 Department of Oral & Maxillofacial Prosthodontics, Graduate School of Biomedical Sciences, Tokushima University, Tokushima 770-8503, Japan; fitri.rahmitasari@gmail.com (F.R.); c301551014@tokushima-u.ac.jp (K.K.); matsuda.takashi.1@tokushima-u.ac.jp (T.M.); megwat@tokushima-u.ac.jp (M.W.); ichi@tokushima-u.ac.jp (T.I.); 2 Department of Dental Material, Faculty of Dentistry, Hang Tuah University, Surabaya 60111, Indonesia

**Keywords:** PEEK, reinforced material, surface modification, dental implant

## Abstract

Polyetheretherketone (PEEK) is a semi-crystalline linear polycyclic thermoplastic that has been proposed as a substitute for metals in biomaterials. PEEK can also be applied to dental implant materials as a superstructure, implant abutment, or implant body. This article summarizes the current research on PEEK applications in dental implants, especially for the improvement of PEEK surface and body modifications. Although various benchmark reports on the reinforcement and surface modifications of PEEK are available, few clinical trials using PEEK for dental implant bodies have been published. Controlled clinical trials, especially for the use of PEEK in implant abutment and implant bodies, are necessary.

## 1. Introduction

Titanium (Ti) and its alloys have been used as dental implants since Brånemark introduced them at the end of the 1960s [1]. Ti materials possess good physicochemical characteristics, mechanical properties, biocompatibility, and high resistance to fatigue stress and corrosion [2,3]. However, Ti materials have an elastic modulus significantly higher than that of bone (titanium: 110 GPa; cortical bone: 14 GPa), and the difference may result in inadequate stress-shielding, bone resorption, and implant fracture [4,5]. In addition, Ti materials have been implicated in clinical problems, such as occasional metal hypersensitivity and allergies, surface degradation and contamination related to peri-implantitis, and scattered radiation [6]. The metallic appearance of Ti materials may also be problematic, as highly aesthetic restorations are becoming important.

Many researchers have undertaken efforts to develop substitutes for Ti dental implants, such as zirconia [7,8], which has a high elastic modulus and low temperature degradation [9,10]. Polymeric compounds, such as polyetheretherketone (PEEK), have been developed as additional substitutes. PEEK is a semi-crystalline linear polycyclic thermoplastic that was developed in 1978 [11]. It can be applied to materials as a superstructure, implant abutment, or implant body.

This article summarizes the current research of the application of PEEK for dental implants, especially for the improvement of surface and body modifications of PEEK for dental implant applications.

## 2. What Is PEEK?

**Figure 1:**
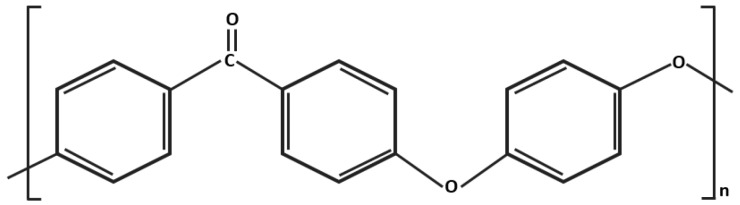
Chemical structure of polyetheretherketone.

PEEK is a dominant of the PAEK (poly-aryl-ether-ketone) polymer family, which has high-temperature stability (exceeding 300 °C) and high mechanical and chemical resistance. It will be a primary substitute for metallic components in the field of orthopedics and trauma [12,13,14,15]. PEEK has an aromatic molecular backbone with combinations of ketone (–CO–) and ether (–O–) functional groups between the aryl rings (Figure 1). PEEK has high stability, low density (1.32 g/cm^3^), insolubility, and a low elastic modulus (3–4 GPa) [16,17].

PEEK has some clinical advantages as a dental implant material compared to Ti. First, it causes fewer hypersensitive and allergic reactions. Certain studies have shown that titanium is an allergen [18]. Second, it is radiolucent and causes fewer artifacts on magnetic resonance imaging [4,19]. Third, it does not have a metallic color; it is beige with a touch of gray, and has a more aesthetic appearance than Ti. Fourth, PEEK is a versatile foundation material that can be tailored to a particular purpose by changing its bulk or surface properties.

PEEK has been applied as an implant material in the implant body, abutment, and superstructure. Applications in the implant body have been limited to bench tests, and there is no report on its application to the mandible as the implant body. If PEEK is used as a dental implant body, it may exhibit lower stress shielding than Ti due to the closer compatibility of the mechanical properties of PEEK and bone [4]. Although PEEK can be applied as a healing abutment or a provisional abutment, no information is available on a final abutment. One method for obtaining the emergence profile in areas around dental implants was shown by Becker (2012), who used a provisional abutment made of PEEK [20]. Koutouzis evaluated soft and hard tissue responses to titanium and provisional PEEK abutments, and reported that no significant difference between PEEK and Ti was found in soft- and hard-tissue responses in 3 the months after the provisional abutment [21]. Another report explains that titanium-reinforced PEEK abutments could be a more effective alternative material compared to conventional titanium abutments, because PEEK can improve the preservation of bone height and soft tissue stability [22]. There is no information on long-term clinical assessment of PEEK abutments; the longest study only lasted several months. Although clinical case reports of PEEK superstructure are available [23], controlled clinical assessment has not yet been reported.

## 3. PEEK Reinforcement

The elastic modulus of PEEK is very low compared to those of cortical bone, Ti, and ceramic materials. The higher elastic modulus of PEEK is required for dental implant materials, especially those used for abutments and superstructures.

Various reinforced PEEK composites have been developed, such as carbon fiber-reinforced PEEK (CFR-PEEK) and glass fiber-reinforced PEEK (GFR-PEEK); the elastic modulus may be as high as 18 GPa for CFR-PEEK [24] and 12 GPa for GFR-PEEK [4]. The elastic modulus of PEEK can also be tailored to closely match the cortical bone or Ti alloy by preparing carbon fiber-reinforced (CFR) composites with varying fiber lengths and orientations. CFR-PEEK has been of interest to the medical implant community due to its versatility, compatibility with modern imaging technologies, excellent mechanical properties, and biocompatibility [25,26]. This material can be manufactured in several shapes with various physical, mechanical, and surface properties [27]. The elastic moduli of the material properties, including reinforced PEEK materials, are shown in Table 1 [4,24,25,28,29,30,31,32,33,34,35,36,37,38].

Based on the energy dissipation theory, a force applied to the implant-supported crown is transferred through the implant, with small alterations due to the energy conservation feature of rigid implants, resulting in an elastic deformation and minimal mechanical energy storage by the implant [39]. Sarot et al. compared the stress distribution of 30% CFR PEEK and Ti using a finite element method (FEM). The findings could lead to the assumption that an endless carbon fiber (stronger CFR-PEEK) dental implant could show decreased stress peaks at the bone-implant interface due to decreased elastic deformation [40]. Based on the results of the above study, Schwitalla et al. compared the biomechanical behavior of three dental implant materials using a FEM: Ti (type 1), powder-filled PEEK (type 2), and Endolign^®^ (type 3), which represented an implantable CFR-PEEK including 60% parallel-oriented endless carbon fibers with an elastic modulus of 150 GPa. Type 2 showed higher von Mises stress peaks and higher maximum deformation, while types 1 and 3 showed similar stress distributions [25]. Lee et al. also compared the compressive strength of GFR-PEEK, CFR-PEEK, and Ti rods. Ti and GFR-PEEK rods showed the highest and lowest compressive strength, respectively [4]. Schwitalla et al. performed static pressure tests with 11 non-reinforced and reinforced PEEK materials and determined that all tested materials were suitable for use as dental implants, based on the compressive force [41].

## 4. Surface Modification of PEEK for Osseointegration

There are many ways in which PEEK can be modified at a nanometer level to overcome its limited bioactivity. Nanoparticles such as TiO_2_, HAF, and HAp can be combined with PEEK through the process of melt-blending to produce bioactive nanocomposites. Moreover, these composites exhibit significantly superior tensile properties when compared to pure PEEK.

Although PEEK has lower osteoconductivity than titanium [42], nanoscale surface modification with hydroxyapatite deposition [43,44], titanium deposition [27], increasing the surface roughness, chemical modifications (sulfonation, amination, and nitration), and incorporation with bioactive properties (TiO_2_ [45,46], hydroxyfluoroapatite [47]) can improve the biocompatibility of PEEK to achieve early osseointegration. Moreover, modified PEEK exhibits significantly superior tensile properties than pure PEEK [48]. PEEK has also been coated with other bioactive materials using plasma spraying [26,44], spin-coating [43,49], plasma gas etching [50], electron-beam deposition [51], and plasma immersion ion implantation [52]. Various surface modifications of PEEK for osseointegration are shown in Table 2 [27,43,44,45,47,49,50,53,54,55,56,57,58,59,60,61,62,63,64,65].

Rust-Dawicki et al. compared the in vivo mechanical strength of the bone interface of titanium-coated and uncoated PEEK dental implants. The thickness of the titanium-coated PEEK implant in this study was 2000 Å applied by plasma vapor deposition to the implant surface. The in vivo experiment was performed on canine femurs. At 4 weeks, the uncoated PEEK dental implants had significantly higher shear strength, but there was no significant difference between the coated and uncoated PEEK implants at 8 weeks. There was no significant difference in bone contact or new bone growth between 4 and 8 weeks in the two groups. At 4 and 8 weeks, the coated specimens had significantly higher percentages of bone contact [27]. Because titanium has potential hypersensitivities in such cases, a titanium coating might affect hypersensitive inflammatory reactions [66]. But, no severe inflammatory response was seen in any specimens, and no interpositionary fibrous tissue was found between the specimens [27].

Unmodified PEEK is a bioinert material, and shows a water-contact angle (CA) of 80–90 degrees, which is close to being a hydrophobic value [64,67,68]. Modified PEEK can have enhanced hydrophilicity, which leads to increased cellular proliferation [69] because the wettability of the biomaterial and the dental implant surface influences the interaction between the material and the surrounding physiological environment [70,71]. The wettability of the dental implant surface can be enhanced by UV irradiation. Qahtani et al. compared the respective changes in wettability of 4 original screw-type implants including PEEK after irradiation with UV-A and UV-C, and reported that the PEEK implants slightly hydrophilized (CA = 79 degrees) during irradiation with UV-C [64].

Xu et al. developed CFR-PEEK-nanohydroxyapatite with micro-/nano-topographical structures by modifying them with oxygen plasma and sandblasting the surface. The aim was to enhance osteogenesis as a potential bioactive material for bone grafting and bone tissue engineering applications with enhanced biocompatibility and osseointegration [60].

## 5. Conclusions

This article reviewed the applications of PEEK in dental implants and the current state of the research. Although various benchmark reports of the reinforcement and surface modifications of PEEK are available, few clinical trials using PEEK for dental implant bodies have been published. Controlled clinical trials, especially for implant abutment and implant bodies, are necessary.

## Figures and Tables

**Table 1:** Elastic moduli of various materials.

Material	Elastic Modulus (GPa)	References
Titanium	110	Lee, 2012 [4]
Cobalt-Chromium	180–210	Wiesli, 2015 [28]
Zirconia	210	Lee, 2012 [4]
Porcelain	68.9	Lewinstein, 1995 [29]
PMMA	3–5	Vallittu, 1998; Zafar, 2014 [30,31]
PEEK	3–4	Sandler, 2002 [24]
CFR-PEEK	18	Sandler, 2002 [24]
Continuous CFR-PEEK (Endolign^®^)	150	Schwitalla, 2015 [25]
GFR-PEEK	12	Lee, 2012 [4]
Cortical bone	14	Martin, 1989; Rho, 1993 [32,33]
Cancellous bone	1.34	Borchers and Reichart, 1983 [34]
Enamel	40–83	Staines, 1981; Rees, 1993; Cavalli, 2004 [35,36,37]
Dentin	15–30	Rees, 1993; Chun, 2014 [36,38]

**Table 2:** Surface modifications of PEEK.

Surface Modifications	Procedures	Material	References
Coating	Plasma spraying	Hydroxyapatite (HA), titanium (Ti)	Rust-Dawicki, 1995; Suska, 2014; Ha, 1994 [27,45,53]
	Spin coating	Nanosized HA crystals containing surfactans, organic solvent, an aquous solution of Ca(NO_3_)_2_ and H_3_PO_4_	Barkarmo, 2012; Johansson, 2014 [43,49]
	Electron-beam evaporation (EBE)	Ti; Silicate	Han, 2010; Wen, 2016 [54,55]
	Plasma immersion ion implantation (PIII)	Titanium dioxide (TiO_2_); calcium (Ca); water (H_2_O); Argon (Ar)	Wang, 2014; Lu, 2014; Lu, 2016; Chen, 2017 [47,56,57,58]
Surface topographical modifications	Acid etching	Sulfuric acid	Zhao, 2013 [59]
	Sandblasting	TiO_2_, alumina (Al_2_O_3_)	Suska, 2014; Xu, 2015 [44,60]
Chemical modifications	Sulphonation	Sulfonate groups (–SO_3_–)	Yee, 2013 [61]
	Amination	Amine functions	Henneuse-Boxus, 1998 [62]
	Nitration	Nitrate functions	Conceição, 2009 [63]
Incorporating with bioactive properties	Bioactive inorganic materials	Nano-TiO_2_ (n-TiO_2_); nano-fluorohydroxyapatite (n-FHA)	Wu, 2012; Wang, 2014 [45,47]
Improving hydrophylicity	UV irradiation	UV-A light, UV-C light	Qahtani, 2015 [64]
	Plasma gas treatment	Oxygen plasma	Waser-Althaus, 2014; Xu, 2015; Poulsson, 2014 [50,60,65]

## References

[1] Brånemark P.I., Adell R., Breine U., Hansson B.O., Lindstrom J., Ohlsson A. (1969). Intraosseous anchorage of dental prostheses. I. Experimental studies. Scand. J. Plast. Reconstr. Surg..

[2] Lautenschlager E.P., Monaghan P. (1993). Titanium and titanium alloys as dental materials. Int. Dent. J..

[3] Renouard F., Nisand D. (2006). Impact of implant length and diameter on survival rates. Clin. Oral Implants Res..

[4] Lee W.T., Koak J.Y., Lim Y.J., Kim S.K., Kwon H.B., Kim M.J. (2012). Stress shielding and fatigue limits of poly-ether-ether-ketone dental implants. J. Biomed. Mater. Res. B Appl. Biomater..

[5] Huiskes R., Ruimerman R., Van Lenthe G.H., Janssen J.D. (2000). Effects of mechanical forces on maintenance and adaptation of form in trabecular bone. Nature.

[6] Schalock P.C., Menné T., Johansen J.D., Taylor J.S., Maibach H.I., Lidén C., Bruze M., Thyssen J.P. (2012). Hypersensitivity reactions to metallic implants-Diagnostic algorithm and suggested patch test series for clinical use. Contact Dermat..

[7] Nakamura K., Kanno T., Milleding P., Ortengren U. (2010). Zirconia as a dental implant abutment material: A systematic review. Int. J. Prosthodont..

[8] Özkurt Z., Kazazoğlu E. (2011). Zirconia dental implants: A literature review. J. Oral Implantol..

[9] Akagi K., Okamoto Y., Matsuura T., Horibe T. (1992). Properties of test metal ceramic titanium alloys. J. Prosthet. Dent..

[10] Kelly J.R., Denry I. (2008). Stabilized zirconia as a structural ceramic: An overview. Dent. Mater..

[11] Eschbach L. (2000). Nonresorbable polymers in bone surgery. Injury.

[12] Fujihara K., Huang Z.M., Ramakrishna S., Satknanantham K., Hamada H. (2003). Performance study of braided carbon/PEEK composite compression bone plates. Biomaterials.

[13] Fujihara K., Huang Z.M., Ramakrishna S., Satknanantham K., Hamada H. (2004). Feasibility of knitted carbon/PEEK composites for orthopedic bone plates. Biomaterials.

[14] Kurtz S.M., Devine J.N. (2007). PEEK biomaterials in trauma, orthopedic, and spinal implants. Biomaterials.

[15] Kurtz S.M. (2012). PEEK Biomaterials Handbook.

[16] Andreiotelli M., Wenz H.J., Kohal R.J. (2009). Are ceramic implants a viable alternative to titanium implants? A systematic literature review. Clin. Oral Implants Res..

[17] Skinner H.B. (1988). Composite technology for total hip arthroplasty. Clin. Orthop..

[18] Manish G., Chandu G., Sunil K.M., Siddharth G. (2014). Titanium allergy: A literature review. Indian J. Dermatol..

[19] Wang H., Xu M., Zhang W., Kwok D.T., Jiang J., Wu Z., Chu P.K. (2010). Mechanical and biological characteristics of diamond-like carbon coated poly aryl-ether-ether-ketone. Biomaterials.

[20] Becker W., Doerr J., Becker B.E. (2012). A novel method for creating an optimal emergence profile adjacent to dental implants. J. Esthet. Restor. Dent..

[21] Koutouziz T., Richardson J., Lundgren T. (2011). Comparative soft and hard tissue responses to titanium and polymer healing abutments. J. Oral Implantol..

[22] Val J.E.M.S.D., Gómez-Moreno G., Martínes C.P.A., Ramírez-Fernández M.P., Granero-Marín J.M., Gehrke S.A., Calvo-Guirado J.L. (2016). Peri-implant tissue behavior around non-titanium material: Experimental study in dogs. Ann. Anat..

[23] Santing H.J., Meijer H.J.A., Raghoebar G.M., Özcan M. (2012). Fracture strength and failure mode of maxillary implant-supported provisional single crowns: A comparison of composite resin crown fabricated directly over PEEK abutments and solid titanium abutments. Clin. Implant Dent. Relat. Res..

[24] Sandler J., Werner P., Shaffer M.S., Demchuk V., Altstädt V., Windle A.H. (2002). Carbon-nanofibre-reinforced poly(ether ether ketone) composites. Compos. Part A Appl. Sci. Manuf..

[25] Schwitalla A.D., Emara M.A., Spintig T., Lackmann J., Müller W.D. (2015). Finite element analysis of the biomechanical effects of PEEK dental implants on the peri-implant bone. J. Biomech..

[26] Najeeb S., Zafar M.S., Khursid Z., Siddiqui F. (2016). Applications of polyetheretherketone (PEEK) in oral implantology and prosthodontics. J. Prosthodont. Res..

[27] Rust-Dawicki A.M., Cook S.D. (1995). Preliminary evaluation of titanium-coated PEEK implants. J. Oral Implantol..

[28] Wiesli M.G., Med M.D., Özcan M. (2015). High-performance polymers and their potential application as medical and oral implant materials: A review. Implant Dent..

[29] Lewinstein I., Banks-Sills L., Eliasi R. (1995). Finite element analysis of a new system (IL) for supporting an implant-retained cantilever prosthesis. Int. J. Oral Maxillofac. Implants.

[30] Vallittu P. (1998). Some aspects of the tensile strength of unidirectional glass fibre-polymethyl methacrylate composite used in dentures. J. Oral Rehabilit..

[31] Zafar M.S., Ahmed N. (2014). Nanoindentation and surface roughness profilometry of poly methyl methacrylate denture base materials. Technol. Health Care.

[32] Martin R., Ishida J. (1989). The relative effects of collagen fiber orientation, porosity, density, and mineralization on bone strength. J. Biomech..

[33] Rho J.Y., Ashman R.B., Turner C.H. (1993). Young’s modulus of trabecular and cortical bone material: Ultrasonic and microtensile measurements. J. Biomech..

[34] Borchers L., Reichart P. (1983). Three-dimensional stress distribution around a dental implant at different stages of interface development. J. Dent. Res..

[35] Staines M., Robinson W., Hood J. (1981). Spherical indentation of tooth enamel. J. Mater. Sci..

[36] Rees J., Jacobsen P. (1993). The elastic moduli of enamel and dentine. Clin. Mater..

[37] Cavalli V., Giannini M., Carvalho R.M. (2004). Effect of carbamide peroxide bleaching agents on tensile strength of human enamel. Dent. Mater..

[38] Chun K.J., Choi H.H., Lee J.Y. (2014). Comparison of mechanical property and role between enamel and dentin in the human teeth. J. Dent. Biomech..

[39] Sheets C.G., Earthmann J.C. (1993). Natural intrusion and reversal in implant assisted prosthesis: Evidence of and a hypothesis for the occurrence. J. Prosthet. Dent..

[40] Sarot J.R., Contar C.M.M., Cruz A.C.C.D., Magini R.S. (2010). Evaluation of the stress distribution in CFR-PEEK dental implants by the three-dimensional finite element method. J. Mater. Sci. Mater. Med..

[41] Schwitalla A.D., Spintig T., Kallage I., Müller W.D. (2016). Pressure behavior of different PEEK materials for dental implants. J. Mech. Behav. Biomed. Mater..

[42] Rabiei A., Sandukas S. (2013). Processing and evaluation of bioactive coatings on polymeric implants. J. Biomed. Mater. Res. Part A.

[43] Barkamo S., Wennerberg A., Hoffman M., Kjellin P., Breding K., Handa P., Stenport V. (2013). Nano-hydroxyapatite-coated PEEK implants: A pilot study in rabbit bone. J. Biomed. Mater. Res. Part A.

[44] Suska F., Omar O., Emanuelsson L., Taylor M., Gruner P., Kinbrum A., Hunt D., Hunt T., Taylor A., Palmquist A. (2014). Enhancement of CRF-PEEK osseointegration by plasma-sprayed hydroxyapatite: A rabbit model. J. Biomater. Appl..

[45] Wu X., Liu X., Wei J., Ma J., Deng F., Wei S. (2012). Nano-TiO_2_/PEEK bioactive composite as a bone substitute material: In vitro and in vivo studies. Int. J. Nanomed..

[46] Wang N., Li H., Lü W., Li J., Wang J., Zhang Z., Liu Y. (2011). Effects of TiO_2_ nanotubes with different diameters on gene expression and ossointegration of implants in minipigs. Biomaterials.

[47] Wang L., He S., Wu X., Liang S., Mu Z., Wei J., Deng F., Deng Y., Wei S. (2014). Polyetheretherketone/nano-fluorohydroxyapatite composite with antimicrobial activity and osseointegration properties. Biomaterials.

[48] Najeeb S., Khurshid Z., Matinlinna J.P., Siddiqui F., Nassani M.Z., Baroudi K. (2015). Nanomodified Peek Dental Implants: Bioactive Composites and Surface Modification—A Review. Int. J. Dent..

[49] Johansson P., Jimbo R., Kjellin P., Currie F., Chrcanovic B.R., Wennerberg A. (2014). Biomechanical evaluation and surface characterization of a nano-modified surface on PEEK implants: A study in the rabbit tibia. Int. J. Nanomed..

[50] Waser-Althaus J., Salamon A., Waser M., Padeste C., Kreutzer M., Pieles U., Müller B., Peters K. (2014). Differentiation of human mesenchymal stem cells on plasma-treated polyetheretherketone. J. Mater. Sci. Mater. Med..

[51] Randolph S., Fowlkes J., Rack P. (2006). Focused, nanoscale electron-beam-induced deposition and etching. Crit. Rev. Solid State Mater. Sci..

[52] Mantese J.V., Brown I.G., Cheung N.W., Collins G.A. (1996). Plasma-immersion ion implantation. MRS Bull..

[53] Ha S., Mayer J., Koch B., Wintermantel E. (1994). Plasma-sprayed hydroxylapatite coating on carbon fibre reinforced thermoplastic composite materials. J. Mater. Sci. Mater. Med..

[54] Han C., Lee E., Kim H., Koh Y., Kim K.N. (2010). The electron beam deposition of titanium on polyetheretherketone (PEEK) and the resulting enhanced biological properties. Biomaterials.

[55] Wen J., Lu T., Wang X., Xu L., Wu Q., Pan H., Wang D., Liu X., Jiang X. (2016). In vitro an in vivo evaluation of silicate-coated polyetheretherketone fabricated by electron beam evaporation. ACS Appl. Mater. Interfaces.

[56] Lu T., Liu X., Qian S., Cao H., Qiao Y., Mei Y., Chu P.K., Ding C. (2014). Multilevel surface engineering of nanostructured TiO_2_ on carbonfiber-reinforced polyetheretherketone. Biomaterials.

[57] Lu T., Qian S., Meng F., Ning C., Liu X. (2016). Enhanced osteogenic activity of poly ether ether ketone using calcium plasma immersion ion implantation. Colloids Surf. B Biointerfaces.

[58] Chen M., Ouyang L., Lu T., Wang H., Meng F., Yang Y., Ning C., Ma J., Liu X. (2017). Enhanced bioactivity and bacteriostatis of surface fluorinated polyetheretherketone. ACS Appl. Mater. Interfaces.

[59] Zhao Y., Wong H.M., Wang W., Li P., Xu Z., Chong E.Y.W., Yan C.H., Yeung K.W.K., Chu P.K. (2013). Cytocompatibility, osseointegration, and bioactivity of three-dimensional porous and nanostructured network on polyetheretherketone. Biomaterials.

[60] Xu A., Liu X., Gao X., Deng F., Deng Y., Wei S. (2015). Enhancement of osteogenesis on micro/nano-topographical carbon fiber-reinforced polyetheretherketone-nanohydroxyapatite biocomposite. Mater. Sci. Eng. C.

[61] Yee R.S.L., Zhang K., Ladewig B.P. (2013). The effects of sulfonated poly(ether ether ketone) ion exchange preparation conditions on membrane properties. Membranes.

[62] Henneuse-Boxusa C., Boxusa T., Dulière E., Pringallea C., Tesolina L., Adriaensenb Y., Marchand-Brynaert J. (1998). Surface amination of PEEK film by selective wet-chemistry. Polymer.

[63] Conceição T.F., Bertolino J.R., Barra G.M.O., Pires A.T.N. (2009). Poly (ether ether ketone) derivatives: Synthetic route and characterization of nitrated and sulfonated polymers. Mater. Sci. Eng. C.

[64] Qahtani M.S.A.A., Wu Y., Spintzyk S., Krieg P., Killinger A., Schweizer E., Stephan I., Scheideler L., Geis-Gerstorfer J., Rupp F. (2015). UV-A and UV-C light induced hydrophilization of dental implants. Dent. Mater..

[65] Poulsson A.H., Eglin D., Zeiter S., Camenisch K., Sprecher C., Agarwal Y., Nehrbass D., Willson J., Richards R.G. (2014). Osseointegration of machined, injection moulded and oxygen plasma modified PEEK implants in a sheep model. Biomaterials.

[66] Schwitalla A., Müller W.D. (2013). PEEK Dental Implants: A Review of the Literature. J. Oral Implantol..

[67] Huang R., Shao P., Burns C., Feng X. (2001). Sulfonation of poly(ether ether ketone) (PEEK): Kinetic study and characterization. J. Appl. Polym. Sci..

[68] Nieminen T., Kallela I., Wuolijoki E., Kainulainen H., Hiidenheimo I., Rantala I. (2008). Amorphous and crystalline polyetheretherketone: Mechanical properties and tissue reactions during a 3-year follow-up. J. Biomed. Mater. Res. Part A.

[69] Wenz L., Merritt K., Brown S., Moet A., Steffee A. (1990). In vitro biocompatibility of polyetheretherketone and polysulfone composites. J. Biomed. Mater. Res..

[70] Rupp F., Gittens R.A., Scheideler L., Marmur A., Boyan B.D., Schwartz Z., Geis-Gerstorfer J. (2014). A review on the wettability of dental implant surfaces I: Theoretical and experimental aspects. Acta Biomater..

[71] Gittens R.A., Scheideler L., Rupp F., Hyzy S.I., Geis-Gerstorfer J., Schwartz Z., Boyan B.D. (2014). A review on the wettability of dental implant surfaces II: Biological and clinical aspects. Acta Biomater..

